# Role of Hsp90 in Systemic Lupus Erythematosus and Its Clinical Relevance

**DOI:** 10.1155/2012/728605

**Published:** 2012-10-04

**Authors:** Hem D. Shukla, Paula M. Pitha

**Affiliations:** ^1^Department of Biology, Johns Hopkins University, Baltimore, MD 21218, USA; ^2^Department of Oncology, Johns Hopkins University School of Medicine, Baltimore, MD 21205, USA

## Abstract

Heat shock proteins (HSP) are a family of ubiquitous and phylogenically highly conserved proteins which play an essential role as molecular chaperones in protein folding and transport. Heat Shock Protein 90 (Hsp90) is not mandatory for the biogenesis of most proteins, rather it participate in structural maturation and conformational regulation of a number of signaling molecules and transcription factors. Hsp90 has been shown to play an important role in antigen presentation, activation of lymphocytes, macrophages, maturation of dendritic cells, and in the enhanceosome mediated induction of inflammation. Systemic lupus erythematosus (SLE) is a chronic autoimmune inflammatory disease with complex immunological and clinical manifestations. Dysregulated expression of Type I interferon **α**, activation of B cells and production of autoantibodies are hallmarks of SLE. The enhanced levels of Hsp90 were detected in the serum of SLE patients. The elevated level of Hsp90 in SLE has also been correlated with increased levels of IL-6 and presence of autoantibodies to Hsp90. This suggests that Hsp90 may contribute to the inflammation and disease progression and that targeting of Hsp 90 expression may be a potential treatment of SLE. The pharmacologic inhibition of Hsp90 was successfully applied in mouse models of autoimmune encephalomyelitis and SLE—like autoimmune diseases. Thus targeting Hsp90 may be an effective treatment for SLE, especially if combined with other targeted therapeutic approaches.

## 1. Introduction

Heat shock proteins (HSPs) are a family of ubiquitous and phylogenically highly conserved proteins and play an essential role as molecular chaperones in protein folding and transport within the cell [1]. These proteins are named according to their molecular weight, which ranges from 17 kDa small HSP families to more than 100 kDa, and they are classified into six families, namely, the HSP100, HSP90, HSP70, HSP60, and HSP40. The classification of HSPs is based on their related functions and sizes (molecular masses). Using the nomenclature adopted after the Cold Spring Harbor Meeting of 1996 [2], family names are written in capitals, for example, HSP70. Major classes of HSPs include the small HSPs, HSP40, 60, 70, 90, and 110. Interestingly, HSP60, 70, and 90 families are the major HSPs implicated in autoimmune diseases, antigen presentation, and innate immunity, Hsp90 is one of the most abundant proteins in the eukaryotic cell. It constitutes up to 1-2% of the cellular protein under physiological conditions, and its expression is several-fold enhanced in response to stress. Under physiological conditions, HSPs exert housekeeping functions and act as molecular chaperones [3, 4] that assist in the proper folding of nascent polypeptide chain preventing their aggregation and misfolding, or as chaperonins that directly mediate protein folding. Moreover, HSPs also play an important role in preventing protein aggregation, degrading unstable and misfolded proteins, and transporting proteins between cellular compartments. Their basal levels facilitate normal protein folding and guard the proteome from the dangers of misfolding and aggregation [5]. Heat shock proteins are expressed both constitutively and under stressful conditions. In addition to the heat shock, a variety of stressful situations, including environmental (ultraviolet radiation or heavy metals), pathological (infections or malignancies), and physiological (growth factors or cell differentiation) stimuli, induce a marked increase in HSP synthesis, a phenomenon known as the stress response [6]. Traditionally, HSPs are regarded as intracellular molecules; however, upon necrotic, but not apoptotic, cell death, HSPs are released into the extracellular compartments [7]. In addition, HSPs can be released extracellularly in response to a number of stressful conditions [8, 9]. The mechanism and the physiological significance of the HSP release independent of necrotic cell death are not clear. However, HSPs are present in circulation of normal individuals [10], and their circulating levels are decreased in aging [11], and increased in a number of pathological conditions [12]. Their increased expression in tissues that are subjected to various proteotoxic stressors (including heat, heavy metals, hypoxia, and acidosis) is an adaptive response that enhances cell survival. Both functions are needed under diseases state. The aim of this paper is to investigate the structure, localization, and regulation of highly conserved HSP90 and its role in SLE by looking for HSP90 containing immune complexes in kidney biopsies of lupus patients. It also describes the role of HSP90 and its family members in etiology of systemic lupus erythematosus and its potential use in designing appropriate therapeutic approaches. 

## 2. Structure, Localization, and Regulation of HSP90

In vertebrates, two distinct genes encode inducible and constitutively expressed isoforms of the protein (HSP90-*α* and HSP90-*β*), but the functional differences between these isoforms are not well understood [13]. Homologues of HSP90 are also found in the endoplasmic reticulum as glucose related protein 94 (GRP94) and in the mitochondria as TNF receptor associated protein 1 (TRAP1). While HSP90 resides primarily in the cytoplasm, one of the HSP90 variant (HSP90N) with a unique hydrophobic N-terminal domain has been found to be a membrane-associated protein. However, its precise cellular function is not clear [14]. The cytoplasmic HSP90 exists predominantly as a homodimer and each homodimer is made up of monomer units which consist of three main domains which are involved in important functional interactions with other cellular targets. The N-terminal domain contains an unusual adenine-nucleotide binding pocket known as the Bergerat fold [15]. The hydrolysis of ATP to ADP in the Bergerat fold has an essential role in the chaperoning activity of the HSP90 dimer. In eukaryotes, a flexible, highly charged linker sequence connects the N-terminal domain to the “middle region” of HSP90. Most molecular chaperones share common functional domains: an adenine nucleotide-binding domain that binds and hydrolyzes ATP and a peptide-binding domain that binds exposed hydrophobic residues of substrate proteins. Binding of ATP triggers a critical conformational change leading to the release of the bound substrate protein [16]. As folding of most newly synthesized proteins in the cell involves interactions with one or more chaperones, protein-binding sites of HSPs by necessity have a broad specificity, and their binding to other cellular proteins is facilitated by hydrophobic interactions [4]. 

Under conditions of stress, such as heat shock, inducible HSPs are highly upregulated by heat shock factors (HSF), which are generated as part of the heat shock response (HSR), to maintain cellular homeostasis and to develop cell survival functions. The heat-shock factors (HSFs) bind to the heat-shock element (HSE) in the promoters of the genes encoding hsps. Four heat shock factors (HSFs) have been identified and well characterized and their roles clearly elucidated. The functional role of HSF1 and HSF3 has been linked to regulating Hsps in response to thermal stress whereas HSF2 and HSF4 are involved in Hsp regulation in unstressed cells and have been linked to a wide variety of biological processes such as immune activation and cellular differentiation [17]. The stresses results in HSF1 oligomerization and nuclear translocalization, followed by enhanced DNA binding on the Hsp gene promoters. It was shown recently that HSF1 is negatively regulated by Hsp90, thus suggesting a negative-feedback loop for the regulation of Hsp90 genes following a heat-shock response [18]. During heat shock response, HSF1 is known to undergo posttranslational modification by various processes including phosphorylation, acetylation, and sumoylation [17]. The HSF2 has also been shown to be bound to the HSE promoter elements of other heat-shock genes, including Hsp90 and Hsp27, as well as the protooncogene c-Fos [19]. These data suggest that HSF2 is important for constitutive as well as stress-inducible expression of HSE-containing genes.

## 3. Role of HSP90 and Its Homologues in Autoimmune Diseases

Infection is a stressful process for both the pathogen and the host and therefore inevitably results in increased production of molecular chaperones by the pathogen as well as by the host. The conservation of HSPs through prokaryotes and eukaryotes, together with the increased production of host and microbial HSPs at the site of infection, suggests that cross-reactivity between host and pathogen HSPs might be responsible for a variety of autoreactive disorders that are associated with high frequency recognition of HSPs [20]. In this context, the possible involvement of mycobacterial HSP70 in the autoantibody production in systemic lupus erythematosus (SLE) has been indicated in one study [21]. 

Autoimmune diseases remain among the most poorly understood and recognized categories of illnesses in the world. Many autoimmune diseases are much more common in women than in men, and estrogens exacerbate systemic lupus erythematosus in murine models of the disease by altering the B-cell repertoire in the absence of inflammation [22]. In addition to serving as molecular chaperones, HSPs have been implicated in autoimmune diseases, antigen presentation, and tumor immunity. Considerable work has also suggested that HSPs, such as Hsp60, Hsp70, Hsp90 and gp96, may be potent activators of the innate immune system capable of inducing the production of proinflammatory cytokines by the monocyte-macrophage system and the activation and maturation of dendritic cells *via *the TLR2- and 4-signal transduction pathways [23]. 

Chaperones function as stimulators of the innate immune system; HSPs have been also shown to play a role in generating antigen-specific T-cell responses [24]. The proposed mechanism is that peptides, complexed with the HSPs including HSP70, Gp96, and calreticulin are delivered to antigen-presenting cells (APCs) by receptor-mediated internalization of the HSPs, making them available for processing and presentation on major histocompatibility complex (MHC) molecules. Specific receptor-mediated mechanisms exist for the capture and internalization of HSPs, suggesting that cross-presentation of HSP-derived antigenic determinants is a legitimate mechanism for cross-priming by professional APCs. Moreover, HSPs can be upregulated in different pathologic conditions and serve as specific targets of the adaptive immune response. Regardless of the participation of HSPs in the pathogenesis of autoimmunity via antigenic cross-reactivity, HSPs are capable of eliciting immune responses [25]. Autoantibodies and cells reactive to HSP have been detected in patients with rheumatoid arthritis, [26], SLE [26] inflammatory bowel disease [27] and multiple sclerosis [28]. The role of this autoimmune response to HSPs in various diseases has not been yet identified. In one case, autoantibodies to HSP90 have been correlated with elevated levels of IL-6 in SLE [12].

Misdirected immune responses target self-antigens and induce severe inflammatory responses, which is a typical sign of autoimmune diseases and sometimes cause death. In addition to numerous components in autoimmune responses, heat shock proteins (HSPs) have been also implicated in autoimmune and inflammatory diseases [29, 30]. Because protein folding is easily impaired by various cytotoxic stresses, such as heat shock, cytotoxic chemicals, hypoxia, and inflammation, prokaryotic to higher eukaryotic organisms have evolved unique mechanism to respond to these stresses. The transcription of HSPs and their subsequent protein expression are stimulated by cytotoxic stresses, and they immediately restore protein folding and cellular homeostasis to counter toxic stresses [5]. Thus, HSPs could be postulated to act as an intracellular protein homeostasis maintenance factors. However, HSPs have also been reported to be observed in the extracellular fluid [11], and act as pro- and anti-inflammatory factors especially in autoimmune and inflammatory diseases in diverse manners. In inflammatory lesions, chaperones are upregulated by inflammatory stress and are released into the extracellular fluid. Subsequently, extracellular HSPs specifically induce proinflammatory cytokines and enhance the antigenicity of autoantigens through modulations of antigen presentation [31, 32]. However, the extracellular HSPs can also stimulate anti-inflammatory regulatory T cell responses, thereby inducing the negative feedback control of inflammation [29, 30]. Indeed, immunization with HSP peptides prevents disease development in autoimmune model animals, such as adjuvant arthritis and collagen-induced arthritis [30]. Presently, besides HSPs, another class of stress proteins is also known to exist in eukaryotic cells known as endoplasmic reticulum (ER) stress proteins, which are specialized factors involved in protein quality control in the secretory pathways [33, 34] are induced by ER stress, which is very different from cytosolic stress [35, 36]. However, similar to HSPs, ER stress proteins are also induced by inflammatory stress [37], suggesting that ER stress proteins might also participate in autoimmune and inflammatory responses. 

## 4. Role of HSP90 in Systemic Lupus Erythematosus (SLE)

Systemic lupus erythematosus (SLE) is a chronic autoimmune inflammatory disease with complex immunological and clinical manifestations. Reduced immune tolerance and abnormal activation of T and B cells lead to autoantibody production mainly against protein-nucleic acid complexes, such as chromatin, and small ribonucleoprotein particles [38]. These autoantibodies complexed with their cognate self-antigens deposit within capillaries of various organs and subsequently mediate systemic disorders [12]. The commonly affected organs include the skin, heart, kidneys, lungs, joints, and central nervous system. SLE is more common in women than in men (>8 : 1). Studies using animal models suggest a role of estrogens in the disease development. The induction of SLE depends on hereditary factors and environmental agents, and inherited genes, infections, ultraviolet light, and some medications are all involved [39]. 

Autoreactivity to HSP is often associated with autoimmune pathology. The investigations have demonstrated the presence of autoantibodies to the HSP90 in a significant proportion of patients with systemic lupus. Anti-HSP90 autoantibodies of the IgG class were detected in approximately 50% [2] and 26% [3], and of IgM class in 35% [40] of patients with SLE. The presence of high concentration of hsp90 autoantibodies was found to correlate with renal disease and low C3 levels [40]. Although HSP90 is an intracytoplasmic protein, surface expression of HSP90 on peripheral blood mononuclear cells was found in approximately 25% of the patients with systemic lupus erythematosus (SLE) during active disease [41]. It has been shown that increased expression of HSP90 is due to the enhanced transcription of the HSP90-alpha gene [5]. While, these results indicate an association of anti-HSP90 autoreactivity with SLE, however, no direct involvement of HSP90 and anti-HSP90 antibodies in the pathogenesis of the disease has been proven. Although the etiology of SLE is still unclear, deposition of antigen-antibody complexes plays a role in the tissue damage of blood vessels and kidney [42]. Autoantibodies of different specificities are found in the sera of patients with SLE. Antibodies to double-strand DNA are found in 52% of the patients with SLE [40], anti-Sm antibodies in 25% [39], and antibodies to components of RNP particles in 25% and 10%, respectively [40]. Anti-HSP90 antibodies were present in 30–50% of the patients with SLE and those patients were more likely to have renal disease and a low C3 level [43]. HSP90 has also been found to be elevated in some subsets of systemic lupus erythematosus (SLE) patients, but its role in the disease is still unknown [39]. Elevated serum levels of HSP90 have been correlated to elevated levels of IL-6 [12] and IL-1. Furthermore, the glomeruli of some SLE patients have been found to have deposits of HSP90 [40]. In addition, HSP90 and its endoplasmic reticulum homologue, glycoprotein 96 (gp96), have also been linked to autoimmunity [44]. In this study the presence of anti-HSP90 autoreactivity was found in 6/10 sera from lupus patients with active disease [40]. The presence of high concentration of HSP90 autoantibodies in the sera of SLE patients might be the most probable cause for the deposition of HSP90 in the kidneys. The increased expression of HSP90, in lupus patients [45], could play an important role as an auto-antigen in the pathogenesis and development of SLE. 

HSP90 is abundant in normal tissues and the relative increase found is difficult to explain the deposits. With these considerations, the results of the present study are in support of the hypothesis that anti-HSP90 autoreactivity is involved specifically in the renal pathology associated with SLE. IgG autoantibodies to HSP90 from SLE patients are found to be less idiotypically regulated within the normal IgG repertoire than natural anti-HSP90 antibodies. These results were supported by the demonstration of an antiidiotypic inhibition of natural anti-HSP90 autoantibodies by IgG antibodies which were more pronounced in normal than in SLE IgG. Thus, the complete IgG repertoire present therein seems to be beneficial in experimental SLE and primary antiphospholipid syndrome [46]. Further, pathogenic anti-HSP90 autoreactivity seems specific SLE, since self-HSP90 participates in the formation of the kidney deposits only in SLE glomerulonephritis [47]. It appears to be a functional expression of the naturally occurring anti-HSP90 IgG autoreactivity due, at least in part, to an altered idiotypic IgG repertoire in sera of SLE patients. The hallmark of SLE is the dysregulation of the immune system, resulting in polyclonal activation of T and B cells, leading to multiorgan inflammation damages from immune complex deposition and infiltrations with inflammatory cells [48]. 

Although lupus-like diseases can be caused by defects in many pathways of the immune system, studies suggest that one of the mechanisms for SLE is the defect in peripheral B- or T-cell tolerance as a result of chronic stimulation and activation of APCs. For example, chronic activation of Langerhans cells by ectopic CD40 ligand expression in the murine epidermis led to not only chronic skin inflammations but also systemic autoimmunity, including immune complex-mediated glomerular nephritis [44]. Activation by genomic DNAs released from dead cells by means of TLR-9 has been implicated in the generation of autoantibodies against Ig [49]. Peripheral monocytes from patients with active, but not quiescent, SLEs were found to differentiate into active DCs more readily, due to higher serum levels of type I IFN [50]. However, whether chronic activations of DCs alone may provoke autoimmunity [51] is still not clear. Chronic activation of B cells, which can be mediated either by activation of TLRs or inflammasomes, certainly contributes to SLE; the association between SNPs in transcription factor IRF-5 (rs 2004640) and predisposition to SLE has also been reported [51].

## 5. Role of HSP90 as Therapeutic Agent for SLE Treatment

Heat shock proteins have been implicated as endogenous activators for dendritic cells (DCs). Chronic expression of heat shock protein gp96 on cell surfaces induces significant DC activations and systemic-lupus-erythematosus- (SLE)-like phenotypes in mice [52]. However, its potential as a therapeutic target against SLE remains to be evaluated. The investigations on chemical approach to determine the role of SLE-like phenotypes could be compromised by controlling surface translocation of gp96. From chemical library screening a compound has been identified that binds and suppresses surface presentation of gp96 by facilitating its oligomerization and retrospective transport to endoplasmic reticulum. [52]. In vivo administration of this compound reduced maturation of DCs, populations of antigen presenting cells, and activated B and T cells. The chemical treatment also alleviated the SLE-associated symptoms such as glomerulonephritis, proteinuria, and accumulation of antinuclear and DNA antibodies in the SLE model mice resulting from chronic surface exposure of gp96. These results suggest that surface translocation of gp96 can be chemically controlled and gp96 is a potential therapeutic target to treat autoimmune disease like SLE. It has also been envisaged that T-cell regulation has long been pursued as a potential therapy for lupus. There are a number of altered or distinct T-cell subtypes in lupus. The DNT cells are known to be upregulated in lupus mice and it is believed that this contributes to the disease state when they infiltrate target organs to produce proinflammatory cytokines and activate B-cell antibody production [51]. Han et al. [52] found that an inhibitor for the endoplasmic reticulum homologue of HSP90, gp96, reduced both the CD44 memory T cells and activated CD44 T cells in the spleen and lymph nodes. The effects of HSP90 inhibition on T-cell populations might be explained by the fact that stimulation of the T-cell receptor leading to T-cell activation requires HSP90 to stabilize lymphocyte-specific protein tyrosine kinase (Lck) in order to initiate activation. Furthermore, it has been shown that HSP90 is an essential regulator for gene expression of linker for activation of T cells (LAT) and that following inhibition of HSP90, both LAT messenger RNA and total LAT protein were decreased. Taken together, HSP90 plays a role in activation of T cells which may be the mechanism behind the changes in T-cell subtypes, found in mice treated with an HSP90 inhibitor. Consequently, targeting HSP90 may be an effective treatment for SLE, especially if combined with other targeted therapeutic approaches [51].

HSP90 has also been reported to play important roles in antigen presentation, activation of lymphocytes and macrophages, and activation and maturation of dendritic cells, indicating a potential treatment target for inflammatory diseases, including autoimmune diseases [7]. This includes patients with systemic lupus erythematosus (SLE) who have elevated levels of Hsp90. Interestingly, elevated levels of circulating IL-6 have also been reported in SLE. Moreover, spontaneous production of IgG by normal and SLE-derived B lymphocytes in culture can be enhanced by the addition of exogenous IL-6 and inhibited by antibody to IL-6. These findings therefore suggest that IL-6 might play a role in the pathogenesis of autoimmune diseases. Moreover, infusion of an antibody to IL-6 can relieve disease symptoms in lupus-prone NZB/NZW mice. Thus, elevated levels of IL-6 in SLE patients induce increased levels of HSP90 protein which in turn results in the production of autoantibodies to this protein. Interestingly, pharmacologic inhibition of Hsp90 has recently been successfully applied in mouse models of autoimmune encephalomyelitis [53], rheumatoid arthritis [54], and systemic lupus erythematosus-like autoimmune diseases [52]. More importantly, in response to stress, HSP90 not only changes its level of expression but may also undergo subcellular redistributions and its trafficking to extracellular milieu, either actively or passively under pathologic stress, is a critical signal for the activation of DCs, which in turn contributes to the initiation and fate determination of adaptive immunity against pathogens as well as self antigens.

## References

[1] Lindquist S (1986). The heat-shock response. *Annual Review of Biochemistry*.

[2] Hightower LE, Hendershot LM (1997). Molecular chaperones and the heat shock response at Cold Spring Harbor. *Cell Stress Chaperones*.

[3] Fink AL (1999). Chaperone-mediated protein folding. *Physiological Reviews*.

[4] Hartl FU, Hayer-Hartl M (2002). Protein folding. Molecular chaperones in the cytosol: from nascent chain to folded protein. *Science*.

[5] Morimoto RI (1998). Regulation of the heat shock transcriptional response: cross talk between a family of heat shock factors, molecular chaperones, and negative regulators. *Genes and Development*.

[6] Pockley AG (2003). Heat shock proteins as regulators of the immune response. *The Lancet*.

[7] Srivastava P (2002). Roles of heat-shock proteins in innate and adaptive immunity. *Nature Reviews Immunology*.

[8] Liao DF, Jin ZG, Baas AS (2000). Purification and identification of secreted oxidative stress-induced factors from vascular smooth muscle cells. *Journal of Biological Chemistry*.

[9] Barreto A, Gonzalez JM, Kabingu E, Asea A, Fiorentino S (2003). Stress-induced release of HSC70 from human tumors. *Cellular Immunology*.

[10] Pockley AG, Bulmer J, Hanks BM, Wright BH (1999). Identification of human heat shock protein 60 (Hsp60) and anti-Hsp60 antibodies in the peripheral circulation of normal individuals. *Cell Stress and Chaperones*.

[11] Njemini R, Bautmans I, Onyema OO, Van Puyvelde K, Demanet C, Mets T (2011). Circulating heat shock protein 70 in health, aging and disease. *BMC Immunology*.

[12] Ripley BJM, Isenberg DA, Latchman DS (2001). Elevated levels of the 90 kDa heat shock protein (hsp90) in SLE correlate with levels of IL-6 and autoantibodies to hsp90. *Journal of Autoimmunity*.

[13] Sreedhar AS, Kalmár É, Csermely P, Shen YF (2004). Hsp90 isoforms: functions, expression and clinical importance. *FEBS Letters*.

[14] Grammatikakis N, Vultur A, Ramana CV (2002). The role of Hsp90N, a new member of the Hsp90 family, in signal transduction and neoplastic transformation. *Journal of Biological Chemistry*.

[15] Dutta R, Inouye M (2000). GHKL, an emergent ATPase/kinase superfamily. *Trends in Biochemical Sciences*.

[16] Csermely P, Schnaider T, Soti C, Prohászka Z, Nardai G (1998). The 90-kDa molecular chaperone family: structure, function, and clinical applications. A comprehensive review. *Pharmacology and Therapeutics*.

[17] Åkerfelt M, Morimoto RI, Sistonen L (2010). Heat shock factors: integrators of cell stress, development and lifespan. *Nature Reviews Molecular Cell Biology*.

[18] Stephanou A, Latchman DS (2011). Transcriptional modulation of heat-shock protein gene expression. *Biochemistry Research International*.

[19] Wilkerson DC, Skaggs HS, Sarge KD (2007). HSF2 binds to the Hsp90, Hsp27, and c-Fos promoters constitutively and modulates their expression. *Cell Stress and Chaperones*.

[20] Zügel U, Kaufmann SHE (1999). Immune response against heat shock proteins in infectious diseases. *Immunobiology*.

[21] Tasneem S, Islam N, Ali R (2001). Crossreactivity of SLE autoantibodies with 70 kDa heat shock proteins of Mycobacterium tuberculosis. *Microbiology and Immunology*.

[22] Pires ES, Choudhury AK, Idicula-Thomas S, Khole VV (2011). Anti-HSP90 autoantibodies in sera of infertile women identify a dominant, conserved epitope EP6 (380–389) of HSP90 beta protein. *Reproductive Biology and Endocrinology*.

[23] Tsan MF, Gao B (2004). Heat shock protein and innate immunity. *Cellular & Molecular Immunology*.

[24] Tamura Y, Peng P, Liu K, Daou M, Srivastava PK (1997). Immunotherapy of tumors with autologous tumor-derived heat shock protein preparations. *Science*.

[25] Murshid A, Gong J, Calderwood SK (2008). Heat-shock proteins in cancer vaccines: agents of antigen cross-presentation. *Expert Review of Vaccines*.

[26] Panchapakesan J, Daglis M, Gatenby P (1992). Antibodies to 65 kDa and 70 kDa heat shock proteins in rheumatoid arthritis and systemic lupus erythematosus. *Immunology and Cell Biology*.

[27] Stevens TRJ, Smith SF, Rampton DS (1993). Antibodies to human recombinant lipocortin-I in inflammatory bowel disease. *Clinical Science*.

[28] Georgopoulos C, McFarland H (1993). Heat shock proteins in multiple sclerosis and other autoimmune diseases. *Immunology Today*.

[29] Hauet-Broere F, Wieten L, Guichelaar T, Berlo S, van der Zee R, Van Eden W (2006). Heat shock proteins induce T cell regulation of chronic inflammation. *Annals of the Rheumatic Diseases*.

[30] Van Eden W, Wick G, Albani S, Cohen I (2007). Stress, heat shock proteins, and autoimmunity: how immune responses to heat shock proteins are to be used for the control of chronic inflammatory diseases. *Annals of the New York Academy of Sciences*.

[31] Multhoff G (2006). Heat shock proteins in immunity. *Handbook of Experimental Pharmacology*.

[32] Yokota SI, Fujii N (2010). Immunomodulatory activity of extracellular heat shock proteins and their autoantibodies. *Microbiology and Immunology*.

[33] Hoseki J, Ushioda R, Nagata K (2010). Mechanism and components of endoplasmic reticulum-associated degradation. *Journal of Biochemistry*.

[34] Araki K, Nagata K (2011). Protein folding and quality control in the ER. *Cold Spring Harbor Perspectives in Biology*.

[35] Mori K (2000). Tripartite management of unfolded proteins in the endoplasmic reticulum. *Cell*.

[36] Walter P, Ron D (2011). The unfolded protein response: from stress pathway to homeostatic regulation. *Science*.

[37] Yoshida H (2007). ER stress and diseases. *FEBS Journal*.

[38] Mahler M, Raijmakers R, Fritzler MJ (2007). Challenges and controversies in autoantibodies associated with systemic rheumatic diseases. *Current Rheumatology Reviews*.

[39] Shimp SK, Chafin CB, Regna NL (2012). Heat shock protein 90 inhibition by 17-DMAG lessens disease in the MRL/lpr mouse model of systemic lupus erythematosus. *Cellular & Molecular Immunology*.

[40] Kenderov A, Minkova V, Mihailova D (2002). Lupus-specific kidney deposits of HSP90 are associated with altered IgG idiotypic interactions of anti-HSP90 autoantibodies. *Clinical and Experimental Immunology*.

[41] Cervera R, Khamashta MA, Font J (2003). Morbidity and mortality in systemic lupus erythematosus during a 10-year period: a comparison of early and late manifestations in a cohort of 1,000 patients. *Medicine*.

[42] Sekine H, Watanabe H, Gilkeson GS (2004). Enrichment of anti-glomerular antigen antibody-producing cells in the kidneys of MRL/MpJ-Faslpr mice. *Journal of Immunology*.

[43] Wu T, Tanguay RM (2006). Antibodies against heat shock proteins in environmental stresses and diseases: friend or foe?. *Cell Stress and Chaperones*.

[44] Liu B, Dai J, Zheng H, Stoilova D, Sun S, Li Z (2003). Cell surface expression of an endoplasmic reticulum resident heat shock protein gp96 triggers MyD88-dependent systemic autoimmune diseases. *Proceedings of the National Academy of Sciences of the United States of America*.

[45] Fenton K, Fismen S, Hedberg A (2009). Anti-dsDNA antibodies promote initiation, and acquired loss of renal dnase1 promotes progression of lupus nephritis in autoimmune (NZB×NZW)F1 Mice. *PloS ONE*.

[46] Levy Y, Sherer Y, Ahmed A (1999). A study of 20 SLE patients with intravenous immunoglobulin—clinical and serologic response. *Lupus*.

[47] Manderson AP, Botto M, Walport MJ (2004). The role of complement in the development of systemic lupus erythematosus. *Annual Review of Immunology*.

[48] Agarwal S, Cunningham-Rundles C (2009). Autoimmunity in common variable immunodeficiency. *Current Allergy and Asthma Reports*.

[49] Christensen SR, Kashgarian M, Alexopoulou L, Flavell RA, Akira S, Shlomchik MJ (2005). Toll-like receptor 9 controls anti-DNA autoantibody production in murine lupus. *Journal of Experimental Medicine*.

[50] Bauer JW, Baechler EC, Petri M (2006). Elevated serum levels of interferon-regulated chemokines are biomarkers for active human systemic lupus erythematosus. *PLoS Medicine*.

[51] Liu Z, Davidson A (2012). Taming lupus-a new understanding of pathogenesis is leading to clinical advances. *Nature Medicine*.

[52] Han JM, Kwon NH, Lee JY (2010). Identification of gp96 as a novel target for treatment of autoimmune disease in mice. *PloS ONE*.

[53] Dello Russo C, Polak PE, Mercado PR (2006). The heat-shock protein 90 inhibitor 17-allylamino-17-demethoxygeldanamycin suppresses glial inflammatory responses and ameliorates experimental autoimmune encephalomyelitis. *Journal of Neurochemistry*.

[54] Rice JW, Veal JM, Fadden RP (2008). Small molecule inhibitors of Hsp90 potently affect inflammatory disease pathways and exhibit activity in models of rheumatoid arthritis. *Arthritis and Rheumatism*.

